# Stimulus-Specific Adaptation at the Synapse Level *In Vitro*

**DOI:** 10.1371/journal.pone.0114537

**Published:** 2014-12-08

**Authors:** Haitao Wang, Yi-Fan Han, Ying-Shing Chan, Jufang He

**Affiliations:** 1 Department of Biomedical Sciences, City University of Hong Kong, Hong Kong, China; 2 Guangzhou Institutes of Biomedicine and Health, Chinese Academy of Sciences, Guangzhou, China; 3 Department of Rehabilitation Sciences, Hong Kong Polytechnic University, Hong Kong, China; 4 Department of Applied Biology and Chemical Technology, Hong Kong Polytechnic University, Hong Kong, China; 5 Department of Physiology and Research Centre of Heart, Brain, Hormone and Healthy Aging, Faculty of Medicine, The University of Hong Kong, Hong Kong, China; Instituto de Neurociencias de Alicante UMH-CSIC, Spain

## Abstract

Stimulus-specific adaptation (SSA) is observed in many brain regions in humans and animals. SSA of cortical neurons has been proposed to accumulate through relays in ascending pathways. Here, we examined SSA at the synapse level using whole-cell patch-clamp recordings of primary cultured cortical neurons of the rat. First, we found that cultured neurons had high firing capability with 100-Hz current injection. However, neuron firing started to adapt to repeated electrically activated synaptic inputs at 10 Hz. Next, to activate different dendritic inputs, electrical stimulations were spatially separated. Cultured neurons showed similar SSA properties in the oddball stimulation paradigm compared to those reported *in vivo*. Single neurons responded preferentially to a deviant stimulus over repeated, standard stimuli considering both synapse-driven spikes and excitatory postsynaptic currents (EPSCs). Compared with two closely placed stimulating electrodes that activated highly overlapping dendritic fields, two separately placed electrodes that activated less overlapping dendritic fields elicited greater SSA. Finally, we used glutamate puffing to directly activate postsynaptic glutamate receptors. Neurons showed SSA to two separately placed puffs repeated at 10 Hz. Compared with EPSCs, GABAa receptor-mediated inhibitory postsynaptic currents showed weaker SSA. Heterogeneity of the synaptic inputs was critical for producing SSA, with glutamate receptor desensitization participating in the process. Our findings suggest that postsynaptic fatigue contributes largely to SSA at low frequencies.

## Introduction

Stimulus-specific adaptation (SSA), which is the decline in neuronal response to repeated stimuli but not a novel stimulus, has been demonstrated in humans and animals at multiple spatial and temporal scales [1], [2], [3], [4]. SSA of single neurons may be involved in the encoding of sensory memories [5] by allowing the detection of changes, thereby enabling animals to extract a meaningful signal from background noise [1], [2]. SSA has been reported in most parts of the ascending auditory pathway and auditory cortex [4], [5], [6], [7], [8] as well as subcortical brain regions, including the reticular thalamic nucleus [9], [10], auditory thalamus [5], [11], [12], and inferior colliculus [13], [14], [15], [16]. Physiological studies have revealed a commonality of SSA within components of the auditory pathway, with adaptation of cortical neurons resulting from the accumulation of adaptation throughout the ascending auditory pathway [4], [8], [9]. Because it is difficult to dissociate the individual contributions of different components in the intact brain, however, the cellular and molecular mechanisms of SSA remain largely speculative.

SSA is thought to be generated via intrinsic membrane excitability changes and short-term synaptic depression [1], [8], [17]. Because cultured neurons *in vitro* form recurrent connections that resemble those *in vivo* in terms of synaptic and intrinsic properties [17], [18], we investigated SSA using whole-cell patch-clamp recording techniques in cultured networks of rat cortical neurons. To examine the mechanisms of SSA at the synapse level, we employed an oddball stimulation paradigm with spatially separated electrical or chemical stimuli [5].

## Materials and Methods

All protocols were approved by the Animal Subjects Ethics Subcommittee at The HongKong Polytechnic University.

### Primary Neuron Cultures

Primary cultured cortical neurons were obtained from 18-day-old Sprague-Dawley rat embryos [19]. Briefly, the cerebral cortex was dissected and incubated with 0.25% trypsin at 37°C for 15 minutes. Cells were then mechanically dissociated using a Pasteur pipette with a fire-narrowed tip in culture medium and plated at a low density of 2×10^4^ cells/ml on 35-mm culture dishes pre-coated with poly-L-lysine (10 µg/ml). Cells were maintained in neurobasal/B27 medium containing 0.5 mM glutamine, 100 units/ml penicillin, and 100 µg/ml streptomycin in a humidified environment of 5% CO_2_/95% air at 37°C. Half-changes of medium were done twice weekly.

### Electrophysiological Recording

Whole-cell patch-clamp recordings were obtained at room temperature from cortical neurons 14–21 days after plating [20]. Signals were amplified with a MultiClamp 700B amplifier, digitized with a Digidata 1440, and acquired with pClamp 10 software (Molecular Devices, USA).The bath solution contained (in mM) 145 NaCl, 3 KCl, 2 MgCl_2_, 3 CaCl_2_,10 HEPES, and 10 glucose (pH 7.4 with NaOH, 300 osmol/L). Patch pipettes with resistance between 3–5 MΩ were pulled from borosilicate glass (WPI, USA) with a Sutter-87 puller (Sutter, USA). For voltage-clamp recordings, pipettes were filled with solutions containing (in mM) 130 caesium methanesulfonate, 10 CsCl, 4 NaCl, 1 MgCl_2_, 10 HEPES, 5 EGTA, 2 QX-314, 2 MgATP, and 0.2 Na-GTP (pH 7.2 with CsOH, 285 osmol/L). For current-clamp and dual patch-clamp recordings, pipettes were filled with solutions containing 136.5 K-gluconate, 0.2 EGTA, 10 HEPES, 9 NaCl, 17.5 KCl, 4 Mg-ATP, and 0.3 Na-GTP (pH 7.2 with KOH, 285 osmol/L).

**Figure 1:**
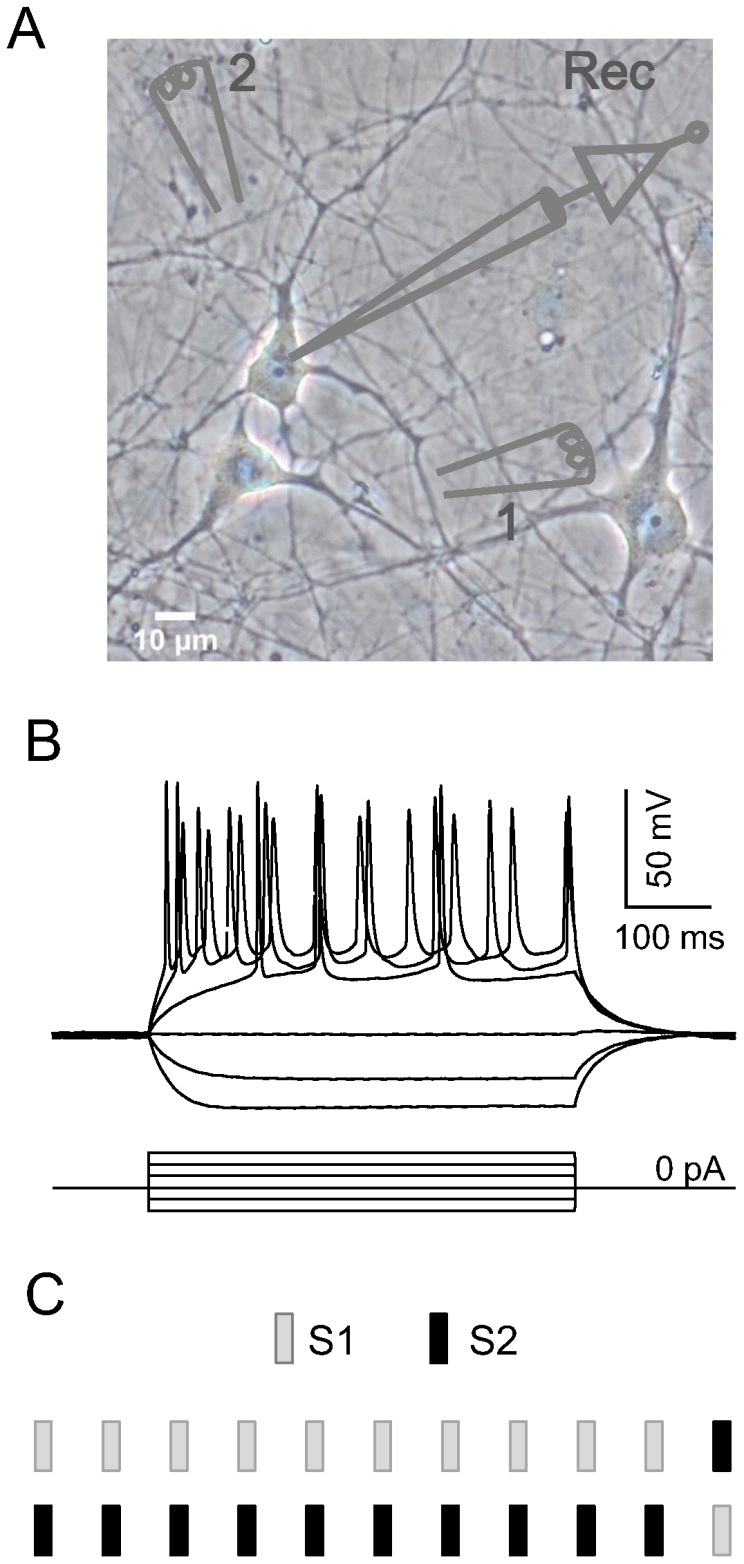
Electrode configuration and experimental paradigm. A, Schematic drawing depicting the electrode configuration: two spatially separated bipolar stimulating electrodes activated different synaptic inputs of the recorded neuron (1: stimulating electrode 1 (S1); 2: stimulating electrode 2 (S2); Rec: recording electrode). B, Characteristic firing pattern of a cortical excitatory neuron in response to step current injection with an increment of 100 pA. C, Oddball stimulation paradigm. Standard and deviant stimuli had a probability ratio of 10∶1, and both orders of stimulus presentation were used in all experiments.

Electrical current stimuli (250-µs duration) were delivered through bipolar stimulating electrodes (FHC, USA) using ISO-Flex stimulus isolators (AMPI, Israel). Electrodes were deliberately positioned near the dish surface to elicit physiological responses, and two different stimulation sites were labeled S1 and S2 (Fig. 1*A*). In the current-clamp mode, synapse-driven spikes were recorded by passing a holding current to maintain neurons at −65 mV, and spikes were counted if the peak voltage exceeded 0 mV. In the voltage-clamp mode, α-amino-3-hydroxy-5-methyl-4-isoxazolepropionic acid (AMPA) receptor-mediated and gamma-aminobutyric (GABA) receptor-mediated currents were recorded by holding neurons at −70 mV or 0 mV, respectively. Pharmacological blockade of AMPA or GABA receptors was achieved by applying 6,7-dinitroquinoxaline-2,3-dione (DNQX, 20 µM) and R-2-amino-5-phosphonopentanoate (APV, 50 µM) or bicuculline (10 µM) and APV (50 µM), respectively, to the bath. Receptor antagonists were purchased from Tocris Cookson Ltd, and other chemicals were purchased from Sigma-Aldrich.

The oddball stimuli in the present study were spatially separated electrical or chemical stimulations. The standard stimuli were the repeated stimulations, and the deviant stimulus was a novel stimulus presented after the standard stimuli. A train of stimuli was applied once every 20 or 30s. The standard and deviant stimuli had a probability ratio of 10∶1, and they were presented in both possible orders in all experiments (Fig. 1*C*) [21].

In the dual patch-clamp experiment, two neurons with a synaptic connection were voltage clamped at −70 mV and recorded simultaneously. Postsynaptic currents were elicited by a 2-ms depolarization of one of the neurons from −70 mV to 40 mV, and the nature of the synaptic connection was ascertained by its dynamics and pharmacological properties. Standard stimuli were 10 stimuli with an inter-stimulus interval (ISI) of 100 ms, and the deviant stimulus was a single stimulus of a different input.

Glutamate puffing was used to examine the SSA of currents mediated solely by postsynaptic glutamate receptors. To directly activate postsynaptic glutamate receptors, glutamate (50 µM) was locally applied with a pressure of 10 psi controlled by a Picospritzer III (Parker, USA). An external solution flowed nearby to reduce receptor desensitization, and neurons were held at −70 mV to record inward ion currents. Two spatially separated drug application electrodes puffed glutamate to different sites of the postsynaptic neuron to activate presumably different groups of glutamate receptors. Using the oddball paradigm, glutamate was puffed at 2, 5, or 10 Hz.

### Data analysis

For the analysis of synapse-driven spikes, success rates of action potentials for standard and deviant stimuli were calculated. For the analysis of postsynaptic currents, the amplitude of responses to each stimulus was normalized to the initial response amplitude. The extent of SSA at each stimulation site (S1 or S2) was quantified using a stimulus index (SI), calculated as SI(Si)  =  [D(Si)-S(Si)]/[D(Si)+S(Si)], (I = 1, 2), where D(Si) and S(Si) were averaged responses to stimulation at Site Si when it was deviant and standard respectively. The ability of neurons to detect the deviant stimulus was assessed using a neuronal index (NI) integrating the SSA effects at the two different sites (S1 and S2), calculated as NI = [D(S1)+D(S2)-S(S1)-S(S2)]/[D(S1)+D(S2)+S(S1)+S(S2)] [4], [5], where D(S1) and S(S1) were averaged responses to stimulation at Site S1 when presented as deviant and standard respectively, and likewise for S2.

Off-line data analysis was performed using Clampfit 10.2 (Molecular Devices, USA). Processed data were imported into Origin 8.0 (OriginLab Corporation, USA) for generating graphs. Numerical data were reported as mean ± SE (standard error), and *P*<0.05 was considered statistically significant.

## Results

Excitatory neurons with a triangular cell body, long dendrites, and a regular spiking pattern (similar to that shown in Fig. 1*B*) were chosen for physiological recordings. The input resistance of the recorded neurons was 202.76±12.70 MΩ (n = 28). Both voltage-clamp and current-clamp recordings were used to examine SSA at the synapse level.

### Neuronal and synaptic adaptation

**Figure 2:**
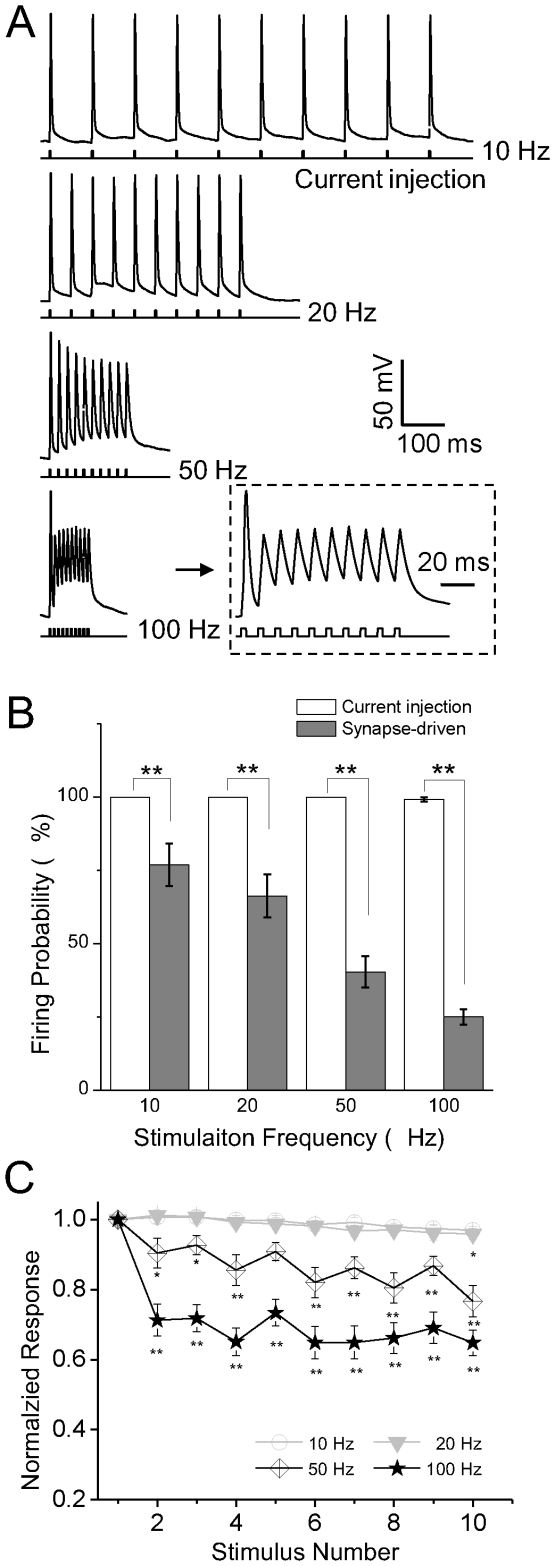
Adaptation of neuronal intrinsic excitability. A, Characteristic neuronal response to intracellular current injection at varying frequencies. Ten pulses were injected during each trial in the current-clamp mode. Insets show responses in expanded time scale. B, Firing probability at varying stimulation frequencies (instristic firing: n = 29; synapse-driven firing: n = 16). C, Normalized spike amplitude plotted against stimulus number. * *P*<0.05, ** *P*<0.01.

First, we examined current-evoked firing capabilities of primary cultured cortical neurons. Single action potentials were elicited by a 3-ms intracellular injection of depolarizing current, and 10 sequential action potentials were evoked with ISIs of 100, 50, 20 or 10 ms to examine firing capabilities at different stimulation frequencies (Fig. 2*A*). At 10 and 20 Hz, all neurons reliably showed phase-locked action potentials (i.e., no adaptation) considering both measures of firing probability (100%; n = 29) and normalized spike amplitude. High firing probability was also observed at 50 Hz (100%; n = 29) and 100 Hz (99.0±0.7%; n = 29). At 100 Hz, however, the amplitude of the 10th spike was reduced to 64.9±3.6% (*P*<0.01; n = 29; ANOVA) of the amplitude of the first spike. These findings indicate that membrane excitability was unchanged at stimulation frequencies less than 100 Hz.

**Figure 3:**
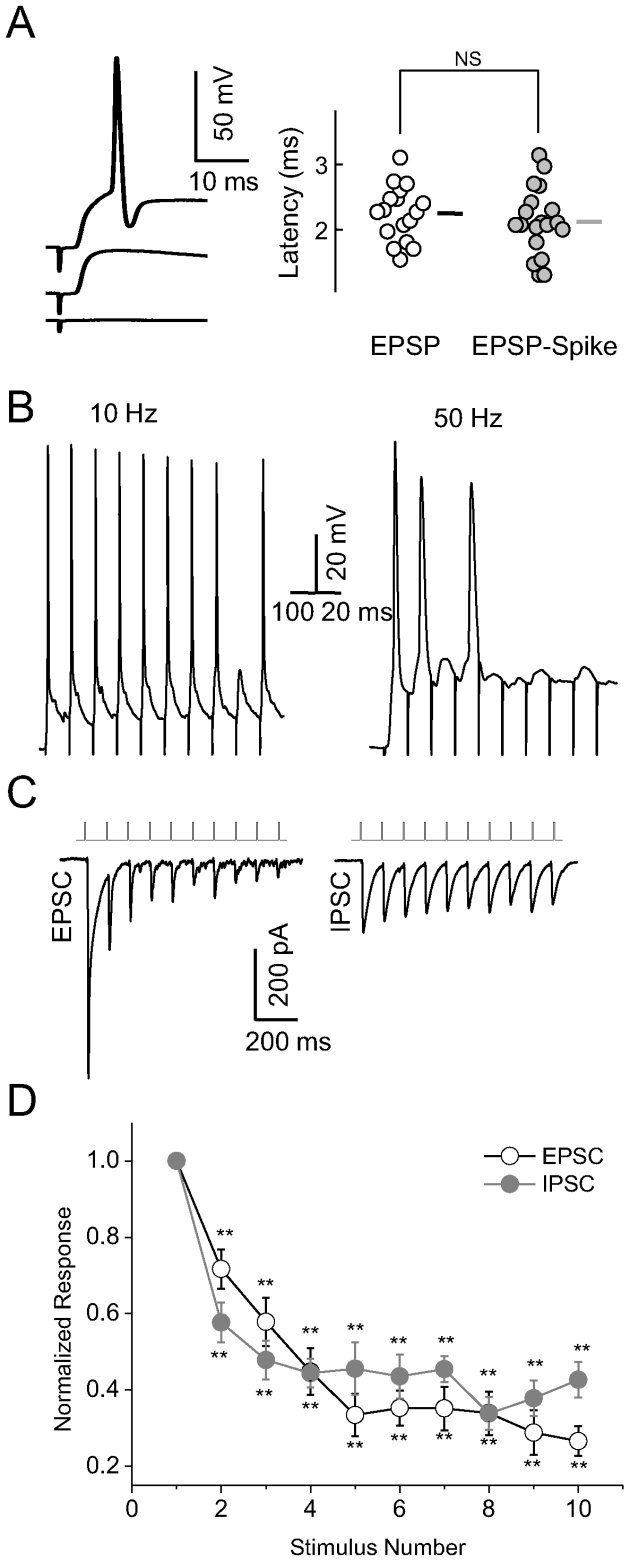
Adaptation of synapse-driven spikes and postsynaptic currents. A, Synapse-driven spikes were elicited with a similar latency as EPSPs. B, Representative traces showing the adaptation of synapse-driven spikes in response to extracellular current stimulation. C, Sample traces of an EPSC (left) and IPSC (right) from two pairs of cortical neurons stimulated at 10 Hz and recorded using dual patch-clamp techniques. D, Normalized postsynaptic current amplitude plotted against stimulus number (n = 27 for EPSC and n = 14 for IPSC).

Next, we investigated adaptation to repeated stimulation at the synapse level using four stimulation frequencies (10, 20, 50, and 100 Hz). Extracellular current stimulation was used to induce presynaptic glutamate release, so that when the postsynaptic membrane potential reached firing threshold, the recorded neuron produced an action potential, which was referred to as a synapse-driven spike (Fig. 3*A*). We found no difference in latency to excitatory postsynaptic potential (EPSP) versus latency to spike (*P*>0.05; n = 29; Student t-test; Fig. 3*A*), indicating their common synaptic origin. As shown in Fig. 3*B*, neurons exhibited 9 action potentials in response to 10 stimulus at 10 Hz, but when stimulation frequency was increased to 50 Hz, only three spikes was observed. There were significant differences in firing probability between current injection and extracellular stimulation conditions at all stimulation frequencies (*P*<0.01 in all cases; Student t-test; Fig. 2*B*), with higher stimulation frequencies associated with greater differences between conditions.

This difference in the probability of current-evoked firing versus synapse-driven spikes suggests that adaptation occurred at the synapse level. Therefore, we performed dual patch-clamp recordings of two neurons to examine adaptation of synaptic transmission. As shown in Fig. 3*C*, in response to artificial pre-synaptic stimulation at 10 Hz, both excitatory postsynaptic currents (EPSCs, left panel) and inhibitory postsynaptic currents (IPSCs, right panel) gradually adapted to repeated stimulation. Compared to the first evoked response, each subsequent response was significantly reduced (*P*<0.01 for stimulus number 2 through 10; n = 27 for EPSCs and n = 14 for IPSCs; ANOVA), indicating that neurons adapted at the synaptic level.

### Stimulus-specific adaptation of synapse-driven spikes

**Figure 4:**
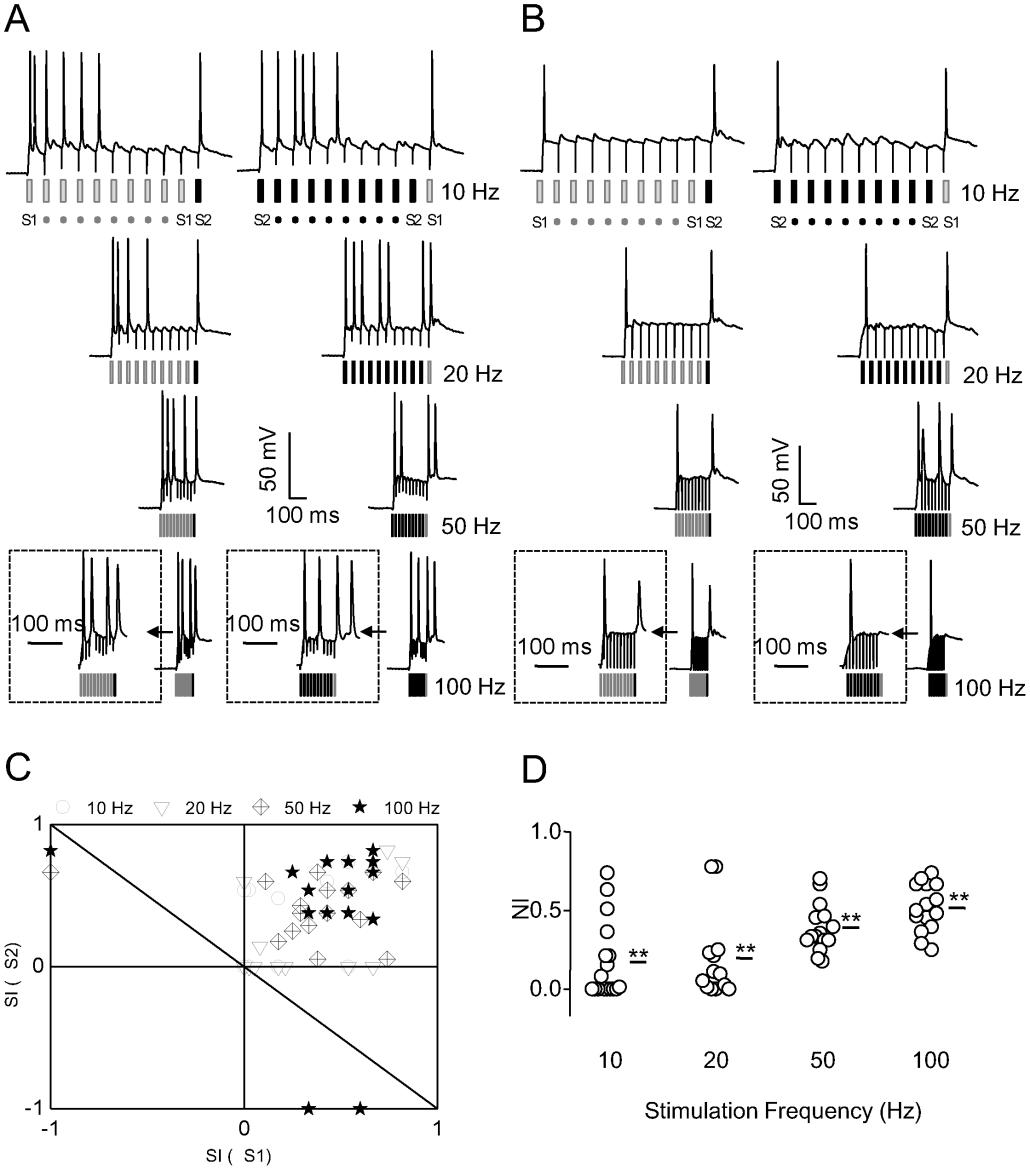
Selective adaptation of synapse-driven spikes. A and B, Characteristic neuronal spikes in response to extracellular stimulation at varying frequencies for a typical (A) and extreme (B) neuron in the oddball paradigm. Note that the sequence of stimulation was reversed in the right panel. Insets show responses expanded in time scale. C, Scatter plot showing SI(S1) against SI(S2) for varying stimulation frequencies (n = 17, 13, 16, and 15 for 10, 20, 50 and 100 Hz, respectively). The diagonal line represents the non-specific bounds, with data points above the line considered as evidence of SSA. D, Individual and average NIs.

Our next question was whether this adaptation was stimulus specific. In other words, we examined whether SSA similar to that recorded *in vivo* also exists at the suprathreshold level *in vitro*. We used the oddball paradigm with two separate stimulating electrodes aimed at different synapses. At 10 Hz, the neuron shown in Fig. 4*A* responded with two spikes to the first electrical stimulus at S1, one spike to each of the subsequent four stimuli, no spike to the next five stimuli, and one spike to the deviant stimulus at S2. After S1 and S2 were reversed, the neuron responded with spikes to the first five stimuli at S2, no spikes to the next five stimuli, and one spike to the deviant stimulus at S1. The same neuron showed a similar response pattern at 20 Hz, but a gradual decrease in response to the standard stimuli was observed at higher frequencies. For instance, at 100 Hz, the neuron showed only three and two spikes in response to repeated stimulation at S1 and S2, respectively, but responded to the deviant stimulus at both sites. Regardless of stimulation frequency, the neuron consistently responded to the deviant stimulus.

SSA indices at the two stimulation sites (SI(S1) and SI(S2)) are shown in Fig. 4*C*. When the stimulation frequency was 100 or 50 Hz, most neurons showed SSA (Fig. 4*C*, data points deviated from the diagonal line, *P*<0.01; n = 16 at 50 Hz and n = 15 at 100 Hz; Signed-rank test). When the frequency was 20 or 10 Hz, however, some neurons showed SSA, but others did not (Fig. 4*C*, data points close to the diagonal line). For example, the neuron shown in Fig. 4*B* exhibited high SSA at 10 Hz, whereas other neurons showed no SSA at this stimulation frequency (data not shown). The SSA effects at the two sites were integrated to compute a neuronal SSA index (NI). Individual NIs and the average NI of synapse-driven spikes at different stimulation frequencies are shown in Fig. 4*D*. The NI for synapse-driven spikes was significantly greater than 0 at all stimulation frequencies (*P*<0.01 at 10, 20, 50, and 100 Hz; Student t-test).

### Stimulus-specific adaptation of synaptic transmission

**Figure 5:**
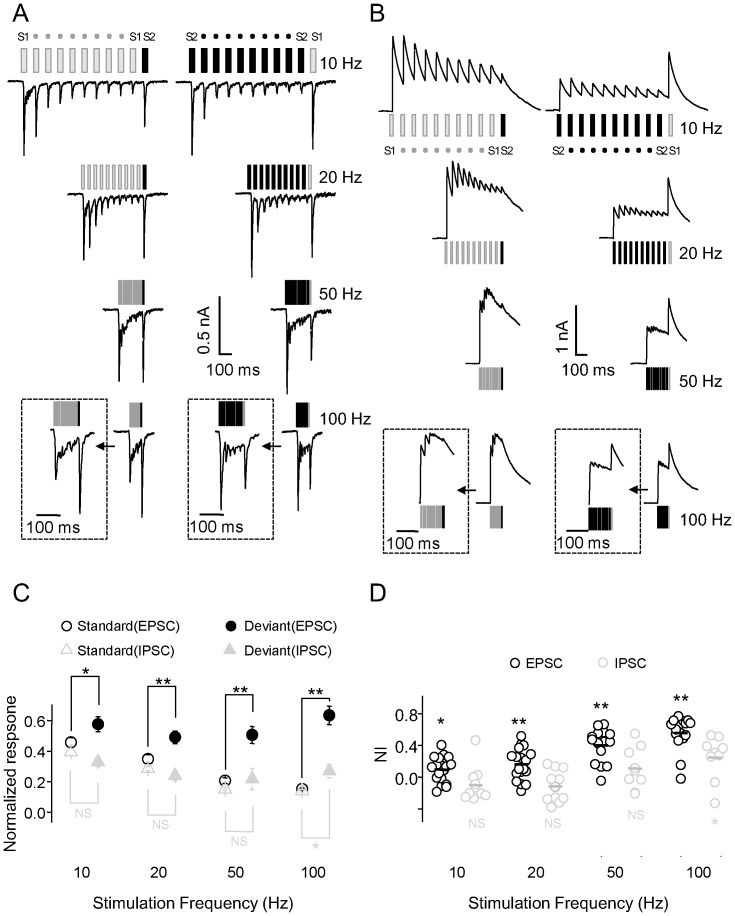
Selective adaptation of synaptic transmission. A and B, Characteristic neuronal EPSCs (A) and IPSCs (B) in response to extracellular electrical stimulation at varying frequencies in the oddball paradigm. Insets show responses expanded in time scale. C, Normalized EPSCs (n = 18, 18, 16 and 16) and IPSCs (n = 11, 11, 9 and 9) in response to standard stimuli compared with the deviant stimulus. D, Individual and average NIs for EPSCs and IPSCs.

Next, we examined whether SSA would be reflected in membrane currents at the subthreshold level. Fig. 5*A and 5B* depict two representative recordings showing EPSCs and IPSCs in response to extracellular currents at different sites.

The neuron shown in Fig. 5*A* exhibited a large EPSC in response to the first standard stimulus and the deviant stimulus at all stimulation frequencies. At 10 and 20 Hz, EPSCs decreased progressively in response to subsequent presentations of the standard stimulus. At 50 or 100 Hz, subsequent EPSCs were barely detectable. Adaptation of responses to repeated stimulation was observed at all frequencies (*P*<0.01 in all cases; n = 18, 18, 16, and 16 for EPSCs and n = 11, 11, 9, and 9 for IPSCs at 10, 20, 50, and 100 Hz, respectively; ANOVA). The amplitude of EPSCs in response to the second presentation of the standard stimulus decreased to 28.9±5.0% (*P*<0.01; n = 18) at 10 Hz and 87.6±3.3% (*P*<0.01; n = 16) at 100 Hz within the same 10-pulse train. However, large EPSCs in response to the deviant stimulus were observed at all frequencies. Normalized EPSCs in response to the deviant stimulus were significantly larger than those to standard stimuli at 50 and 100 Hz (*P*<0.01; n = 16; Student t-test; Fig. 5*C*). Neuronal NIs for EPSCs increased with higher stimulation frequencies and were significantly greater than 0 at all frequencies (*P*<0.05 at 10 Hz, *P*<0.01 at 20, 50, and 100 Hz; Student t-test; Fig. 5*D*). SI(S1) + SI(S2) was also significantly greater than 0 at higher frequencies (*P*<0.05 at 10 Hz, *P*<0.01 at 20, 50, and 100 Hz; Signed-rank test; data not shown).

We also examined SSA of inhibitory inputs. The neuron shown in Fig. 5*B* exhibited a large IPSC in response to the first standard stimulus at S1, with a gradual decline in response amplitude upon subsequent stimulations (Fig. 5*B*, left panel at 10 Hz). The neuron also responded to the first standard stimulus at S2 (although the response was smaller than that at S1), again with a gradual decline in response amplitude upon subsequent stimulations (Fig. 5*B*, right panels at 10 Hz and 20 Hz). Only at 100 Hz was the NI for IPSCs significantly greater than 0 (*P*<0.05 at 100 Hz, *P*>0.05 at 10, 20, and 50 Hz; Student t-test; Fig. 5*D*). Normalized IPSCs in response to the standard and deviant stimuli were equivalent except at stimulation frequency of 100 Hz (*P*<0.05 at 100 Hz, *P*>0.05 at 10, 20, and 50 Hz; Student t-test; Fig. 5*C*).

### Three-site stimulations and dual patch-clamp experiments

In the previous experiments, the two stimuli were spatially separated, theoretically targeting inputs of different synaptic origin. In the next experiment, we used three stimulating electrodes, with the third electrode (S3) placed close to S2 but far from S1. The stimulation paradigm consisting of S1 and S2 was termed as less overlapping condition, while that consisting of S2 and S3 as highly overlapping condition.

**Figure 6:**
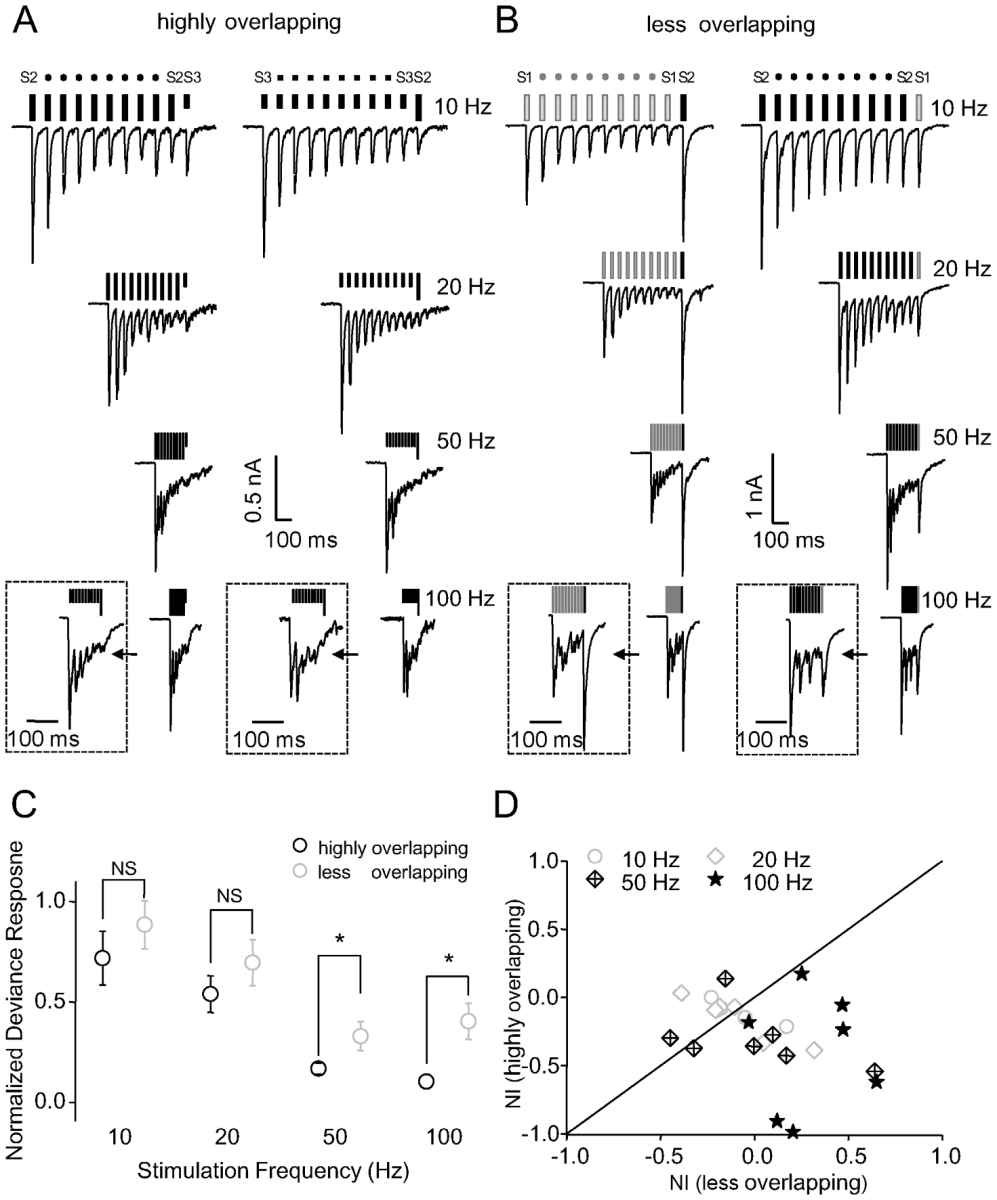
Three-site stimulation to examine identity of synaptic input. A and B, Characteristic neuronal EPSCs in response to extracellular electrical stimulation at varying frequencies for highly overlapping (A) and less overlapping (B) conditions in the oddball paradigm. Placement of stimulating electrodes was modified from that shown in Fig. 1A. Electrodes S1 and S2 were separately placed to activate less overlapping areas, whereas electrodes S2 and S3 were closely placed to activate highly overlapping areas. Insets show responses expanded in time scale. C, Comparison of normalized responses to the deviant stimulus between less overlapping and highly overlapping conditions (n = 5, 6, 7, and 7 for 10, 20, 50, and 100 Hz, respectively). D, NIs for EPSCs in highly overlapping and less overlapping conditions. The diagonal line represents the equiprobable line, with most points biased toward the less overlapping condition.

Adaptation of responses occurred not only to the standard stimuli but also to the deviant stimulus in the highly overlapping condition (Fig. 6*A*), when the two stimulation sites (S2 and S3) were close together. This was in sharp contrast to the less overlapping condition (Fig. 6*B*), in which the neuron responded strongly to the deviant stimulus when the two stimulation sites (S1 and S2) were far apart. EPSCs in response to the deviant stimulus in the less overlapping condition were significantly larger than those in the highly overlapping condition at higher stimulation frequencies (*P*<0.01 at 50 and 100 Hz; n = 7; Fig. 6*C*). The location of most NIs for EPSCs below the diagonal line in Fig. 6*D* suggests that greater SSA occurred in the less overlapping condition than in the highly overlapping condition, with a significant difference between conditions at 100 Hz (*P*<0.01 at 100 Hz, *P*>0.05 at all other frequencies; Signed-rank test).

**Figure 7:**
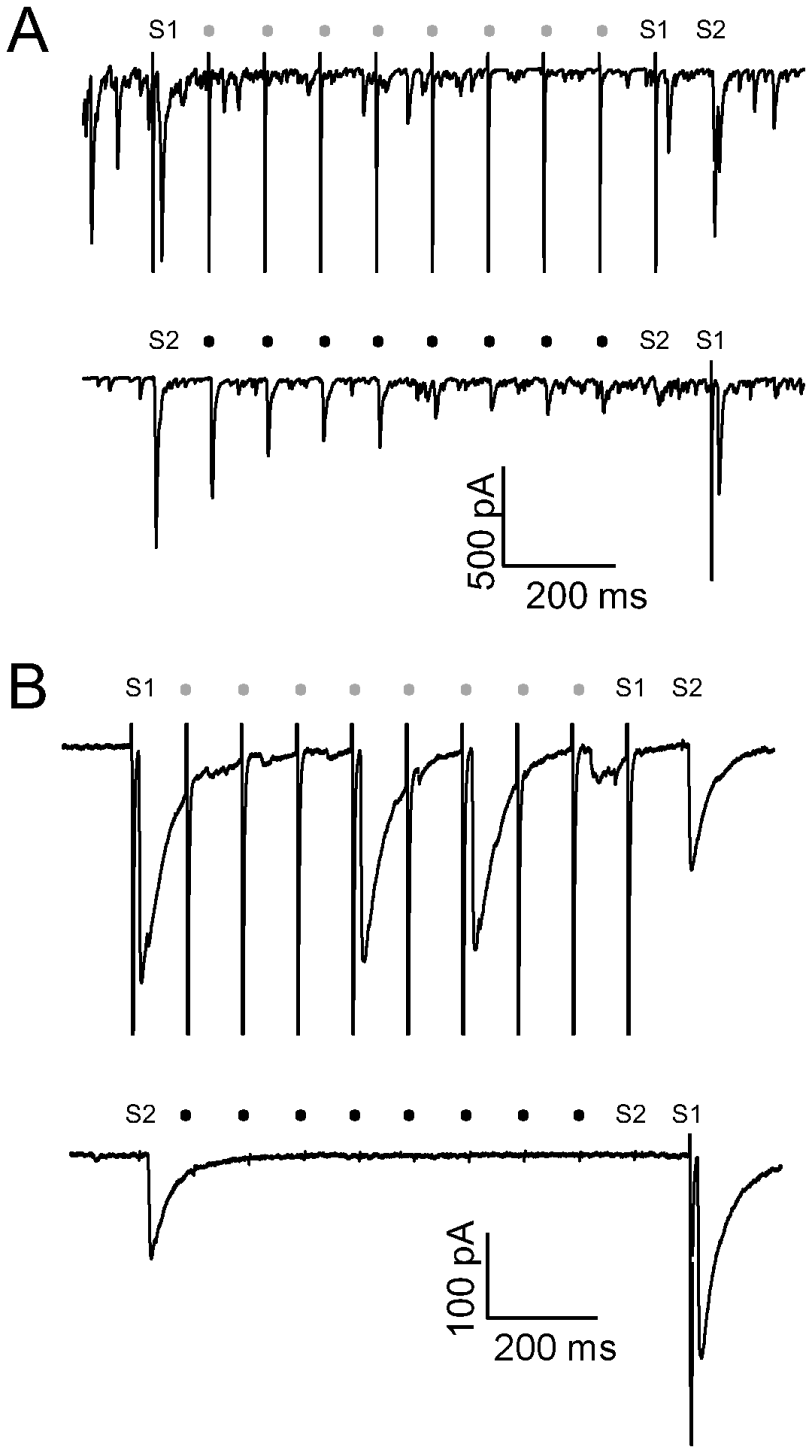
SSA of EPSCs and IPSCs measured by dual patch-clamp recording. A and B, Representative traces showing SSA of an EPSC (A) and IPSC (B) at a stimulation frequency of 10 Hz. Postsynaptic currents were elicited by activation of auto-synaptic or trans-neuronal synaptic inputs. EPSCs adapted to repeated auto-synaptic stimuli, but a robust response reoccurred following the deviant trans-neuronal synaptic stimulus (A, upper panel). When the order of stimuli was reversed (A, lower panel), EPSCs adapted to repeated trans-neuronal synaptic stimuli, but a robust response reoccurred following the deviant auto-synaptic stimulus. This SSA profile was also found for IPSCs recorded from two inhibitory neurons (B).

Next, we employed dual patch-clamp recording to more accurately activate well-separated synaptic inputs. Using the oddball paradigm, auto-synaptic input served as one stimulus, and trans-neuronal synaptic input served as the other stimulus. Prominent SSA of EPSCs (Fig. 7*A*) and IPSCs (Fig. 7*B*) was observed at 10 Hz. EPSCs adapted to the repeated auto-synaptic stimuli (S1) but were large upon delivery of the deviant trans-neuronal stimulus (S2) (Fig. 7*A*, upper panel). Similarly, EPSCs adapted to the repeated trans-neuronal stimuli (S2) but were large upon delivery of the deviant auto-synaptic stimulus (S1) (Fig. 7*A*, lower panel). Similar results were obtained with two inhibitory neurons (Fig. 7*B*).

### Stimulus-specific adaptation of glutamate-activated currents

**Figure 8:**
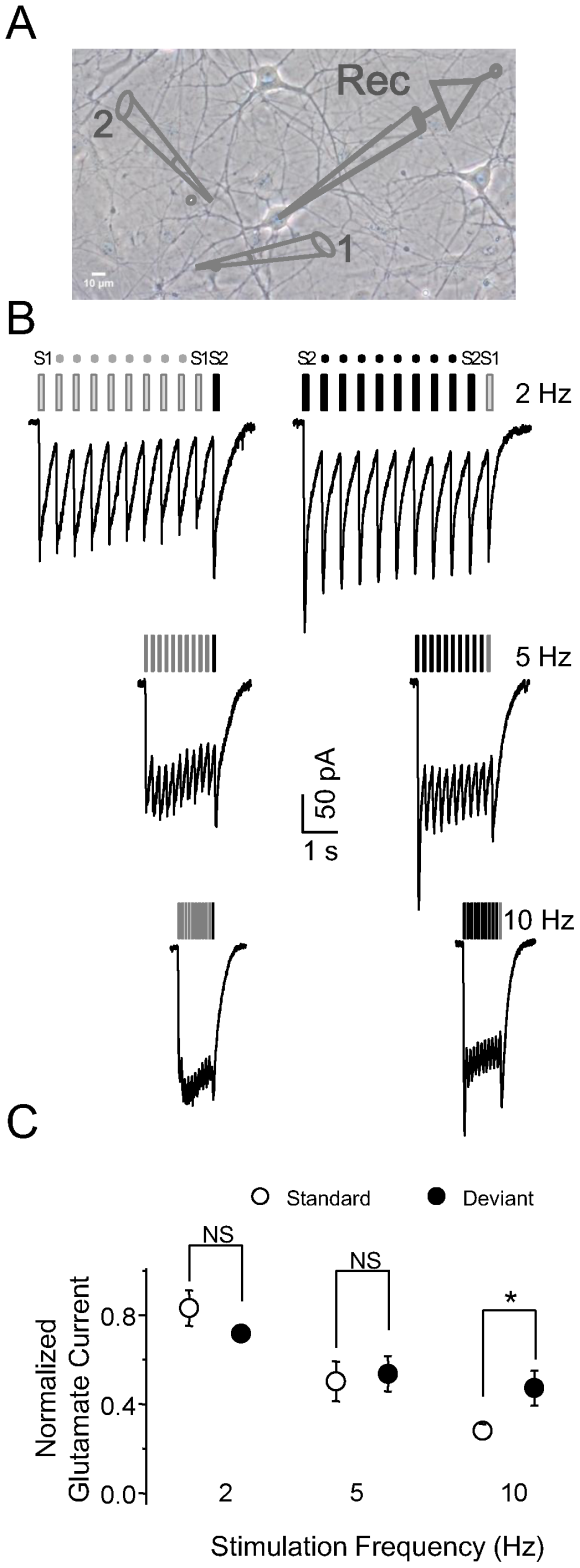
Selective adaptation of glutamate puff-induced currents. A, Schematic drawing depicting the electrode configuration: two spatially separated electrodes filled with glutamate activated different groups of receptors on the recorded neuron (1: glutamate electrode 1 (S1); 2: glutamate electrode 2 (S2); Rec: recording electrode). Glutamate puffs at the two different sites activated different groups of postsynaptic glutamate receptors. B, Characteristic responses to glutamate puffs at varying frequencies. C, Comparison of normalized responses to glutamate-activated current between standard stimuli and the deviant stimulus (n = 4).

SSA at the synapse level could occur at either the presynaptic terminal or the postsynaptic dendrite. Therefore, our next question was whether neurotransmitter release to the receptors would lead to adaptation of postsynaptic responses. Two spatially separated drug pipettes were used to pressure-puff glutamate on two different sites of the neuron, thereby directly activating receptors on the postsynaptic membrane (Fig. 8*A*). The longer duration of glutamate-activated currents made it impossible to repeat stimuli with short ISIs, therefore glutamate puffs were pressure-injected at frequencies of 2, 5, and 10 Hz. Adaptation of glutamate-activated currents to repeated standard stimuli was apparent at 2 Hz (Fig. 8*B*, right panel) and become more pronounced at 5 and 10 Hz. Glutamate-activated currents in response to puffs at the deviant site were significantly greater than those at the standard site at 10 Hz (*P*<0.05 at 10 Hz, *P*>0.05 at 2 and 5 Hz; n = 4; Student t-test; Fig. 8*C*). The stimulus-specific adaptation of glutamate-activated currents occurred only at postsynaptic sites, which underpinned the role of AMPA receptors in the process of adaption at lower stimulation frequency.

## Discussion

We found that *in vitro* cultured cortical neurons exhibited SSA at the synapse level similar to that reported in the *in vivo *brain. Our major findings were: (1) Cultured neurons had high firing capability with100 Hz stimulation and even higher firing capability with current injection, but neuronal firing started to adapt to electrically activated synaptic inputs at 10 Hz. (2) Single neurons preferentially showed synapse-driven spikes and EPSCs in response to a deviant stimulus as compared with standard stimuli. (3) Two separately placed stimulating electrodes that activated less overlapping dendritic fields elicited more SSA than two closely placed electrodes that activated highly overlapping dendritic fields. (4) Neurons showed SSA to two separately placed glutamate puffs repeated at 10 Hz.

Previous studies have found that SSA of extracellular spikes, local field potentials, and blood oxygen consumption signal occurs in cortical and sub-cortical brain regions [1]. SSA to acoustic stimuli is found in the auditory cortex [4], [5], [7], [22], reticular thalamic nucleus [9], medial geniculate body [12], [23], [24], and inferior colliculus [13], [14], [15], [16], [25] in a variety of species (rat, mouse, cat, bat, and barn owl) in anesthetized or awake states. The ability of reticular thalamic nucleus neurons to detect deviant stimuli can either enhance or suppress medial geniculate body neuronal responses to subsequent auditory stimuli [9], and this novelty detection is thought to encode sensory memories [5]. Network interactions, such as intra-cortical processing, have been suggested to account for SSA in both auditory and visual cortices [5], [26]. Intra-cortical mechanisms generate a laminar difference in SSA among cortical layers [7], and projection patterns and stereotyped neural circuitry impacts the SSA properties of target nuclei [9], [12].

Recording from single neurons in a simplified preparation of cultured cortical neurons suggests that SSA is a common feature of the nervous system. Similar to *in vivo* situations, we observed that the selective adaptation of synapse-driven spikes in single cultured neurons was dependent on stimulation frequency, with an NI of 0.52 at 100 Hz but an NI of only 0.17 at 10 Hz. The low density of cultured neurons in the present study made it possible to record monosynaptic spikes and to clearly detect SSA. This suggests that in addition to complex brain structures, simple neuronal connections can also generate SSA but within a narrower temporal window, with ISIs of 10, 20, 50, or 100 ms in our cultured neurons compared with 375, 1000, or 2000 ms in the intact brain [4], [5], [27]. This discrepancy could be caused by the differences in experimental techniques and preparations, and also by the fact that the amount of SSA of cortical neurons in intact animals is accumulated through multiple stations along the auditory ascending pathways, but that of cortical neurons in culture is accomplished between monosynaptic connections.

Intrinsic adaptation of a neuron results in a global reduction of excitability regardless of the stimulated pathways, and activity-dependent activation of Na^+^- and Ca^2+^-mediated potassium conductance contributes to the frequency adaptation of cortical neuron firing [17], [28]. Our results show that cortical neurons sustained firing up to 100 Hz in response to intracellular current stimulation, but synapse-driven spikes started to adapt at 10 Hz. Therefore, the adaptation observed in our neurons cannot be ascribed to intrinsic adaptation but instead may be due to synaptic depression, agreeing to previous result from barrel cortical neurons in response to whisker stimulations [21].

SSA of auditory brain structures has been observed with various stimulation parameters such as sound frequency, amplitude, and duration, with sound frequency being used most frequently [5], [9], [22]. Different frequencies of sound excite different sensory hair cells within the cochlea [29], and frequency separation is maintained throughout upstream auditory stations [30], [31]. In *in vivo* experiments, two different synaptic inputs to auditory neurons were activated by sounds in the oddball paradigm, and the observed SSA could be underpinned by changes in either presynaptic or postsynaptic neurons. Short-term depression of EPSCs possibly functions as a gain control mechanism to favor optimal neural coding [32], [33], [34], and, as expected, we observed reduced excitatory synaptic transmission in response to repeated standard stimuli but an enhanced response to a deviant stimulus. Even at rather low frequencies, EPSCs showed pronounced depression in our dual patch-clamp and extracellular stimulation experiments.

SSA of EPSCs had a similar profile to that of synapse-driven spikes, suggesting that adaptation of synaptic efficacy caused the changes observed at the suprathreshold spike level. High levels of SSA in subthreshold membrane potentials experienced by auditory cortex neurons highly correlated with those of tone-evoked spikes [35], which was consistent with our *in vitro* findings. A stable brain state requires a balance between excitation and inhibition [36]. The larger NI for excitatory response versus the smaller NI for inhibitory response probably helps to produce obvious NI for synapse-driven spikes, as augmented excitation and/or reduced inhibition lowers the threshold for spiking, and hyperpolarization can offset the excitation brought on by excitatory synaptic inputs. This data clearly explains why NI for synapse-driven spikes is more significant than those for excitatory synaptic transmission, which was also confirmed *in vivo* [35]. We observed less depression of IPSCs compared with EPSCs. It is logically reasoned that a loss of inhibition lessens the extent of sensory SSA. GABA-mediated inhibition was recently found to enhance SSA in inferior colliculus neurons in anesthetized rats [15], [37] and cultured cortical neurons [17]. GABA receptor-mediated changes in conductance could contribute to adaptation within a time scale of 150 ms [38]. Apart from inhibition, the inherent neuronal properties also weight SSA, as auditory neurons of cochlear nuclei did not express SSA in sharp contrast with the auditory neurons of the midbrain and forebrain [27], and these nuclei are well characterized by the high expression of Kv3.1 channels and the presence of “endbulb of Held” [39].

Short-term synaptic plasticity is comprised of two components: presynaptic transmitter release and postsynaptic receptor activation [40]. At the presynaptic terminal, high frequency stimulation could exhaust neurotransmitter-containing vesicles [41], and at the postsynaptic membrane, the continuous presence of glutamate could desensitize AMPA receptors [42]. To examine the contribution of postsynaptic components in SSA, we used glutamate puffing to directly activate postsynaptic AMPA receptors. SSA of glutamate-activated currents was similar to that of EPSCs. Neurons showed differences in normalized glutamate-activated currents between standard stimuli and the deviant stimulus at a stimulation frequency of 10 Hz. These results indicate that postsynaptic receptors contribute to the generation of SSA when stimulation frequency is low. When stimulation frequency is high, it is likely that presynaptic components are involved.

**Figure 9:**
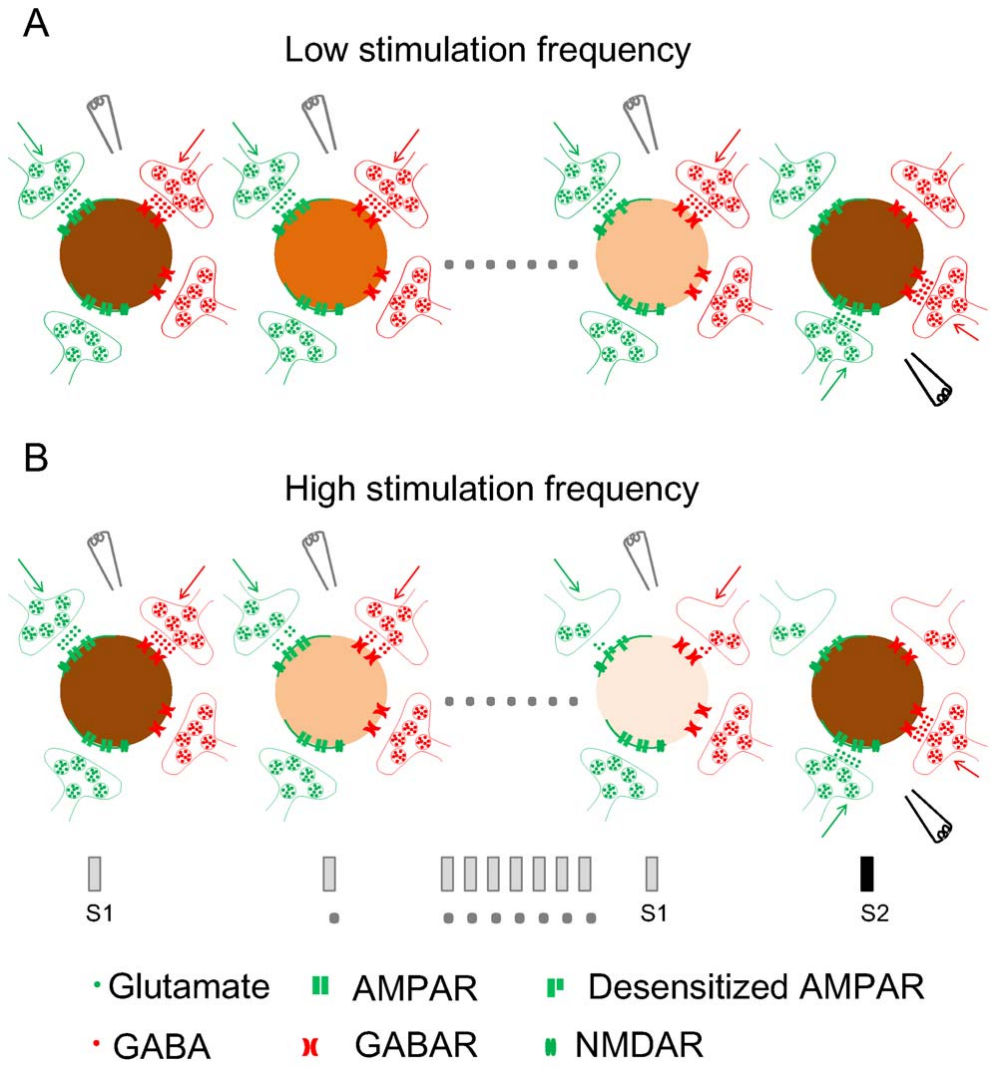
Proposed model of SSA at the synapse level. In response to low frequency stimulation, presynaptic neurons continuously release neurotransmitters, and the remaining glutamate in the synaptic cleft desensitizes AMPA receptors (AMPARs), resulting in a diminished response to the same amount of glutamate upon subsequent stimulation (A). In response to high frequency stimulation, presynaptic vesicles are depleted of neurotransmitter, compromising neuronal response to subsequent stimuli (B). Neuronal response to repeated stimulation at S1 gradually declines, but response to stimulation of a different synaptic origin at S2 is not influenced by activation history because both presynaptic terminals and postsynaptic receptors at this site are naïve. Thus, SSA appears to result from synaptic depression expressed mostly at the postsynaptic site at low stimulation frequencies but at both presynaptic and postsynaptic sites at high stimulation frequencies.

The greater SSA in the less overlapping condition than in the highly overlapping condition in our three-site experiment, together with the prominent SSA observed in our dual patch-clamp experiment, suggests that the identity of synaptic inputs is critical for SSA. We propose a model illustrating SSA at the synapse level for a single neuron (Fig. 9). Two stimuli are delivered in oddball order, with each activating different groups of presynaptic neurons. In response to repeated stimulation, presynaptic neurons continuously release neurotransmitters. With low stimulation frequency, the remaining glutamate in the synaptic cleft desensitizes AMPA receptors (AMPARs), resulting in a diminished response to the same amount of glutamate released upon subsequent stimulations (Fig. 9*A*). With high stimulation frequency, presynaptic glutamate and GABA vesicles are depleted (Fig. 9*B*), compromising neuronal response to repeated stimulation. Neuronal response to repeated stimulation at S1 gradually declines, but response to stimulation of a different synaptic origin at S2 is not influenced by activation history because both presynaptic terminals and postsynaptic receptors at this site are naïve. Thus, SSA appears to result from synaptic depression expressed mostly at the postsynaptic site at low stimulation frequencies but at both presynaptic and postsynaptic sites at high stimulation frequencies. Cortical neurons normally function with firing rates between 0.6 and 16 Hz [8], which is quite different from hippocampal neurons, which sustain firing rates at 100 Hz or higher to serve roles in memory processes [43]. Therefore, postsynaptic fatigue may largely contribute to the SSA in cortical neurons. Other potential mechanisms, such as inhibition mediated by GABAb and metabotropic glutamate receptors, are likely to contribute a great deal to SSA, but are not addressed in this model. However we should carefully generalize our model to sensory SSA. Firstly, lack of totality of the neural circuit, young cortical neurons, and the non-specific effect of bicuculline on potassium channels made the conditions of our experiment different from those conducted *in vivo* [44]. Secondly, our model probably mimics frequency discrimination *in vivo*, but auditory cortex neurons in intact animals still have obvious SSA even at fine frequency difference [35]. Finally, other sound properties such as duration and interaural time/level difference produce SSA [11], [22], indicating other mechanisms accounting for the generation of SSA. Although technical limitations with our model exist, our findings at least provided direct cellular evidence that the imbalance of excitation and inhibition might contribute to SSA at the synapse level.

Taken together, our results provide evidence that, *in vitro*, repeated stimulation causes SSA via synaptic depression. Differences between SSA of EPSCs and IPSCs could underlie SSA of synapse-driven spikes. Our findings suggest that synapse-specific fatigue is a candidate neural mechanism of SSA. As cortical neurons typically function at low frequencies, postsynaptic components may largely contribute to SSA.
